# Vaccination Coverage for Routine Vaccines and Herd Immunity Levels against Measles and Pertussis in the World in 2019

**DOI:** 10.3390/vaccines9030256

**Published:** 2021-03-13

**Authors:** Pedro Plans-Rubió

**Affiliations:** 1 Department of Health of Catalonia, Public Health Agency of Catalonia, 08005 Barcelona, Spain; pedro.plans@gencat.cat; 2 Ciber of Epidemiology and Public Health (CIBERESP), 28028 Madrid, Spain

**Keywords:** routine vaccines, vaccination coverage, anti-measles herd immunity, anti-pertussis herd immunity, WHO regions, vaccination program performance

## Abstract

In 2012, the World Health Organization (WHO) established the Global Vaccine Action Plan with the objective to promote essential vaccinations in all countries and achieve at least 90% vaccination coverage for all routine vaccines by 2020. The study assessed the mean percentages of vaccination coverage in 2019 for 13 routine vaccines, vaccination coverage variation from 2015 to 2019, and herd immunity levels against measles and pertussis in 2019 in countries and regions of WHO. In 2019, the mean percentages of vaccination coverage were lower than 90% for 10 (78.9%) routine vaccines. The mean percentages of vaccination coverage also decreased from 2015 to 2019 for six (46.2%) routine vaccines. The prevalence of individuals with vaccine-induced measles immunity in the target measles vaccination population was 88.1%, and the prevalence of individuals with vaccine-induced pertussis immunity in the target pertussis vaccination population was 81.1%. Herd immunity against measles viruses with Ro = 18 was established in 63 (32.5%) countries but not established in any region. Herd immunity against pertussis agents was not established in any country and in any region of WHO. National immunization programs must be improved to achieve ≥90% vaccination coverage in all countries and regions. Likewise, it is necessary to achieve ≥95% vaccination coverage with two doses of measles vaccines and three doses of pertussis vaccines in all countries and regions.

## 1. Introduction

In 1974, the World Health Organization (WHO) established the Expanded Program on Immunization with the objective to promote essential vaccinations in all countries of the world [1]. The Bacillus Calmette–Guérin (BCG) vaccine against tuberculosis, the diphtheria-tetanus-pertussis (DTP) vaccine, the polio vaccine, and the measles-containing vaccine (MCV) were included in the immunization program [1]. Since 2014, the hepatitis B vaccine, the *Haemophilus imfluenzae* vaccine, the pneumococcal conjugated vaccine, and the rotavirus vaccine have been included in the Expanded Program on Immunization [2].

In 2012, WHO proposed the Global Vaccine Action Plan 2011–2020 [2], with the following objectives: (1) achieve a world free of poliomyelitis; (2) meet vaccination coverage objectives in every region, country, and community; (3) reduce child mortality; (4) meet global and regional elimination objectives; and (5) develop and introduce new and improved vaccines.

The Global Vaccine Action Plan (GVAP) was approved by the World Health Assembly to achieve the Decade of Vaccines vision by delivering universal access to immunization [2]. The mission outlined in the GVAP is to improve health by extending by 2020 and beyond the full benefits of immunization to all people, regardless of where they are born, who they are, or where they live.

It is expected that the Global Vaccine Action Plan 2011–2020 will prevent 20 million deaths from measles, 5.3–6 million deaths from hepatitis B, 1.4–1.7 million deaths from *Haemophilus imfluenzae* infections, 1.6–1.8 million deaths from pneumococcal infections, 0.8–0.9 million deaths from rotavirus infections, and 0.4 million deaths from rubella [2]. The Expanded Program on Immunization has reduced the mortality and morbidity due to infectious diseases in all regions [2,3]. Nevertheless, many vaccine-preventable diseases and outbreaks are occurring in countries and regions of WHO [4,5,6,7]. During 2000–2016, the annual reported measles incidence decreased globally, but the measles incidence increased in all regions of WHO during 2017–2019 [5]. In the European Union, 13,200 cases of measles were reported in 2019 [6] and 389 cases of rubella were reported in 2017 [7].

Sustained high percentages of vaccination coverage are the key preventive measure to successfully control and eliminate the infectious diseases included in the Expanded Program on Immunization and the Global Vaccine Action Plan [2,8,9,10]. First, polio, measles, and rubella vaccination programs have eliminated endemic polio, measles, and rubella from countries and regions of the world [2,3,9]. Second, immunizations provide individual protection against vaccine-preventable infectious diseases and herd immunity against vaccine-preventable infections transmitted from person to person.

The WHO and the United Nations Children’s Fund (UNICEF) collect and review the information reported by different countries of the world on their percentages of vaccination coverage. WHO has proposed to achieve percentages of vaccination coverage of at least 90% with three doses of diphtheria-tetanus-pertussis (DTP) vaccines by 2015 and percentages of vaccination of at least 90% with all vaccines in national programs by 2020 [11]. The strategy plan of WHO to achieve measles elimination in Europe is based on achieving and maintaining percentages of vaccination coverage of at least 95% with two doses of measles-mumps-rubella (MMR) vaccines [12,13]. WHO assumes that the percentages of vaccination coverage recommended for routine vaccines could generate and maintain the herd immunity required to block the transmission of infectious agents in the community [12,13,14,15]. The objectives of this study were:

Determine the mean percentages of vaccination coverage for routine vaccines included in the WHO Expanded Program on Immunization in regions of WHO in 2015 and 2019.

1. Assess the variation from 2015 to 2019 in the mean percentages of vaccination coverage for routine vaccines in regions of WHO.
2. Determine the vaccination coverage with one and two doses of measles vaccines in each country and determine the mean percentages of vaccination coverage with one and two doses of measles vaccine in regions of WHO in 2019.
3. Determine the mean percentages of vaccination coverage with one, two, and three doses of pertussis vaccines in each country and determine the mean percentages of vaccination coverage with one, two, and three doses of pertussis vaccines in regions of WHO in 2019.
4. Assess the prevalence of vaccine-induced measles immunity and anti-measles in each country and determine the mean prevalence in regions of WHO in 2019.
5. Assess the prevalence of vaccine-induced pertussis immunity in each country and determine the mean prevalence in regions of WHO in 2019.
6. Assess anti-measles and anti-pertussis herd immunity levels in each country and regions of WHO in 2019.

## 2. Materials and Methods

The analysis was carried out in four phases: (1) to determine the mean percentages of vaccination coverage for routine vaccines in different regions of WHO from country-specific values in 2015 and 2019; (2) to determine the percentages of vaccination coverage with one and two doses of measles vaccines and the percentages of vaccination coverage with one, two, and three doses of DTP vaccines in different countries of the world and regions of WHO; (3) to determine the prevalence of vaccine-induced protection against measles and against pertussis in different countries of the world and regions of WHO; and (4) to determine the establishment of anti-measles and anti-pertussis herd immunity in different countries of the world and regions of WHO.

### 2.1. Mean Percentages of Routine Vaccination Coverage in Regions of WHO in 2015 and 2019

The mean percentages of vaccination coverage in 2015 and 2019 for routine vaccines included in the WHO Expanded Program on Immunization were calculated for different regions of WHO using the information from the WHO–UNICEF global and regional immunization information system [16,17]. The following immunizations were included in the analysis: (1) bacille Calmette–Guérin (BCG) vaccine against tuberculosis; (2) first dose of diphtheria-tetanus-pertussis (DTP1) vaccine; (3) third dose of diphtheria-tetanus-pertussis (DTP3) vaccine; (4) first dose of hepatitis B vaccine; (5) third dose of hepatitis B vaccine; (6) third dose of *Haemophilus influenzae* vaccine; (7) first dose of inactivated poliovirus vaccine; (8) first dose of measles-containing vaccine (MCV1); (9) second dose of measles-containing vaccine (MCV2); (10) third dose of pneumococcal conjugate vaccine; (11) third dose of poliovirus vaccine; (12) first dose of rubella-containing vaccine; and (13) last dose of rotavirus vaccine.

The mean percentages of routine vaccination coverage were determined for different regions of WHO by dividing the sum of percentages of vaccination coverage reported by countries in a region by the number of countries reporting vaccination coverage in that region. The WHO–UNICEF global and regional immunization information system collects information about the vaccination coverage reported by each country for 13 routine vaccines for the nationally recommended age [18]. Reported values for a dose of inactivated poliovirus vaccine begin in 2015 following the global polio eradication strategic plan for 2013–2018, which recommended at least one dose of inactivated poliovirus vaccine [17].

The WHO–UNICEF global and regional immunization information system collects information about routine vaccination. Consequently, vaccines administered in mass vaccination campaigns are not collected by the WHO–UNICEF global and regional immunization information system [18,19]. The percentages of vaccination coverage reported by different countries were obtained by using administrative and survey-based data. Administrative coverage is the number of doses administered to individuals in the target vaccination population divided by the target population. Survey-based coverage is obtained from a representative sample of the population. Survey-based coverage is the number of individuals who receive a vaccine divided by the total sample of individuals from the target vaccination population included in the survey. Usually, a representative sample of individuals from the target vaccination population is obtained using a two-stage sampling method, where a representative sample of households is obtained in the first phase, and in the second phase, vaccination information is obtained from all individuals who should be vaccinated. Vaccination information is obtained using the method of interviewing caregivers of children and reviewing vaccination documents.

### 2.2. Mean Vaccination Coverage with One and Two Doses of Measles Vaccine and Mean Vaccination Coverage with One, Two, and Three Doses of Pertussis Vaccine in Regions of WHO in 2019

The mean percentages of vaccination coverage with one and two doses of measles vaccine and one, two, and three doses of DTP vaccine in different regions of WHO were determined from the vaccination coverage with one and two doses of measles vaccine and one, two, and three doses of DTP vaccine in countries of the region (phase 2 of the analysis). Routine measles vaccination is based on two doses of a measles-containing vaccine (measles-rubella-mumps (MMR) vaccine) administered at 12–15 months (MCV1) and 3 years of age (MCV2) [13,20,21]. The percentages of vaccination coverage with MCV1 and MCV2 reported by different countries to the WHO–UNICEF global and regional immunization information system are provided for the nationally recommended age [18].

The vaccination coverage with two doses of measles vaccine (V_2_) in the target vaccination population was determined for each country from the vaccination coverage with MCV2 in 2019 and the vaccination coverage with MVC1 in 2017:

V_2_ = MCV2 × MCV1. (1)

It was assumed that the target vaccination population vaccinated with MCV2 in 2019 was vaccinated with MCV1 in 2017 [13,21]. The vaccination coverage with one dose of measles vaccine (V_1_) in the target vaccination population was determined for each country using the formula

V_1_ = (MCV1 − V_2_) + (MCV2 − V_2_). (2)

Routine diphtheria-pertussis-tetanus vaccination is based on three doses of DTP vaccine administered at 2, 4, and 6 months and booster doses administered at 15–18 months and 4–6 years of age [22]. The vaccination coverage with three doses of pertussis vaccine (V_3_) in the target vaccination population (children during their first year of life) in 2019 was determined for each country from the vaccination coverage with the first, second, and third doses of diphtheria-tetanus-pertussis (DTP1, DTP2, and DTP3, respectively) vaccines in 2019:

V_3_ = DTP1 × DTP2 × DTP3. (3)

The vaccination coverage with the second dose of DTP (DTP2) vaccine was estimated for each country from the average vaccination coverage with DTP1 and DTP3 vaccines:

DTP2 = (DTP3 + DTP1)/2.(4)

This calculation was necessary because the vaccination coverage with DTP2 vaccine was not reported to the WHO–UNICEF global and regional immunization information system [16]. Routine vaccination against diphtheria, tetanus, and pertussis can use vaccines based on acellular pertussis antigens (DTaP and dTap vaccines) and vaccines based on whole-cell pertussis antigens (wDTP vaccines). Since 1990, most developed countries use acellular pertussis vaccines due to safety concerns associated with whole-cell pertussis vaccines, but whole-cell pertussis vaccines are used in low-income countries [22].

The vaccination coverage with two doses of DTP vaccine (V_2_) was determined for each country using the formula

V_2_ = [(DTP1 × DTP2) − V_3_] + [(DTP1 × DTP3) − V_3_] + [(DTP2 × DTP3) − V_3_] (5)

The vaccination coverage with one dose of DTP vaccine (V_1_) was determined in each country using the formula

V_1_ = (DTP1 − V_3_ − V_2 (DTP1)_) + (DTP2 − V_3_ − V_2 (DTP2)_) + (DTP3 − V_3_ − V_2 (DTP3)_). (6)

In this formula, V_3_ is the vaccination coverage with three doses of DTP vaccine and V_2 (i)_ is the vaccination coverage with two doses of DTP vaccine, including each DTP vaccine (i = DTP1, DTP2, DTP3).

The following indicators of regional vaccination program performance in 2019 were determined in this study using the vaccination coverage with one and two doses of measles vaccine and one, two, and three doses of DTP vaccine in different countries: (1) mean vaccination coverage with two doses of measles vaccine, (2) mean vaccination coverage with three doses of DTP vaccine, (3) mean percentage of children with zero doses of DTP vaccine in the target vaccination population (children during their first year of life), and (4) mean percentage of children with zero doses of measles vaccine in the target vaccination population. The vaccination coverage with two doses of measles vaccine and the vaccination coverage with three doses of DTP vaccine are good indicators of a country’s vaccination program performance because they show the percentage of children in the target vaccination population who have completed the two-dose measles vaccination and three-dose DTP vaccination, respectively. The mean regional values of these parameters are good indicators of regional vaccination program performance because they show the mean percentage of children who have completed the two-dose measles vaccination and three-dose DTP vaccination in countries of the region. The percentage of children with zero doses of DTP vaccine is equal to 100 minus the vaccination coverage with one, two, and three doses of DTP vaccine:

100 − V1_DTP_ − V2_DTP_ − V3_DTP_. (7)

The percentage of children with zero doses of measles vaccine is equal to 100 minus the vaccination coverage with one and two doses of measles vaccine:

100 − V1_MRM_ − V2_MRM_. (8)

The indicator based on the vaccination coverage with two doses of measles vaccine has been determined in other studies [20,23]. Indicators based on the vaccination coverage with DTP1 and DTP3 vaccines were considered in other studies [19]. The indicator based on vaccination coverage with three doses of DTP vaccine has not been assessed in previous studies.

### 2.3. Herd Immunity Levels against Measles and Herd Immunity Levels against Pertussis in the Target Vaccination Population in Countries of the World and Regions of WHO in 2019

Anti-measles and anti-pertussis herd immunity levels in the target vaccination populations in countries of the world and regions of WHO in 2019 were assessed by (1) determining the prevalence of vaccine-induced protection against measles and pertussis in the target vaccination populations in different countries of the world (phase 3 of the analysis) and (2) assessing whether mean regional values were higher or lower than the critical prevalence associated with herd immunity for different measles and pertussis agents, respectively (phase 4 of the analysis).

The prevalence of individuals with vaccine-induced measles protection in the target measles vaccination populations was determined for each country using the formula

I_v_ = (V_1_ × E_1_) + (V_2_ × E_2_). (9)

In this formula, V_1_ and V_2_ are the percentages of vaccination coverage with one and two doses of the vaccine, respectively, and E_1_and E_2_ are the effectiveness in preventing measles cases with one and two doses of the vaccine, respectively.

The vaccination coverage with one dose of measles vaccine (V_1_) was determined using the formula

V_1_ = (MCV1 − V_2_) + (MCV2 − V_2_). (10)

Effectiveness values of 92% and 95% in preventing measles cases were assumed in this study for one and two doses of measles vaccine, respectively [24,25,26].

The prevalence of individuals with vaccine-induced pertussis protection in the target pertussis vaccination populations was determined for each country using the formula

I_v_ = (V_1_ × E_1_) + (V_2_ × E_2_) + (V_3_ × E_3_). (11)

In this formula, V_1_, V_2_, and V_3_ are the percentages of vaccination coverage with one, two, and three doses of DTP vaccine, respectively, and E_1_, E_2_, and E_3_ are the effectiveness in preventing pertussis cases with one, two, and three doses of DTP vaccine, respectively. Effectiveness values of 84%, 77%, and 59% in preventing pertussis cases were assumed in this study for three, two, and one dose of pertussis vaccines, respectively. The Cochrane Collaboration review obtained an efficacy of 84–85% for three or more doses of acellular pertussis vaccines and 59–78% for one and two doses of acellular vaccines in preventing typical whooping cough (characterized by 21 or more consecutive days of paroxysmal cough with confirmation of *Bordetella pertussis* infection by culture, appropriate serology, or contact with a household member who has culture-confirmed pertussis) [27]. The effectiveness assumed in this study for three doses of pertussis vaccines was 84% because this effectiveness was obtained in the study developed by Greco et al. [28] including 14,751 children over an average of 17 months. Effectiveness values of 59% and 77% in preventing pertussis cases were assumed in this study for one and two doses of pertussis vaccine, respectively, based on the relative effectiveness obtained for one and two doses of pertussis vaccines compared to three doses of the vaccine [28,29]. The same effectiveness was assumed in this study for different doses of acellular and whole-cell pertussis vaccines because the Cochran review concluded that acellular pertussis vaccines with three or more components are more effective than low-efficacy whole-cell vaccines but may be less effective than the highest-efficacy whole-cell vaccines [27].

Herd immunity is defined as the indirect protection of susceptible individuals brought about by the presence of immune individuals in the population. The generation of epidemics depends on the average number of individuals directly infected (secondary cases) by one infectious case during the entire infectious period, when the infectious agent has entered a totally susceptible population [30]. This number is called the basic reproductive number R_o_. The critical prevalence of protected individuals required to establish herd immunity against measles and pertussis (I_c_), determined using the formula

I_c_ = [1 − (1/R_o_)], (12)

is equal to 94.4% for infectious agents, with a value of R_o_ equal to 18. Anderson and May found values of R_o_ ranging from 12 to 18 for measles and from 10 to 18 for pertussis in the review carried out in 1991 [30]. A recent study found values of R_o_ of up to 45 for measles viruses [20,31].

Vaccination programs can block measles and pertussis transmission in the community when the prevalence of individuals with vaccine-induced protection (I_v_) is higher than the critical prevalence associated with herd immunity (I_v_ > I_c_) [32].

In this study, herd immunity in the target population vaccination against measles viruses with R_o_ values of 10, 12, 15, 18, 19, and 20 were assessed in countries and regions of WHO. Herd immunity was considered established against measles viruses with R_o_ values of 10, 12, 15, 18, 19, and 20 when the prevalence of individuals with vaccine-induced measles protection was higher than 90%, 91.7%, 93.3%, 94.4%, 94.7%, and 95%, respectively. Herd immunity levels against pertussis agents with R_o_ values of 10, 12, 15 and 18 were assessed in countries and regions of WHO. Herd immunity was considered established against pertussis agents with R_o_ values of 10, 12, 15, and 18 when the prevalence of individuals with vaccine-induced pertussis protection was higher than 90%, 91.7%, 93.3% and 94.4%, respectively.

### 2.4. Statistical Analysis

Microsoft Excel (Microsoft Corporation, Redmond, WA, USA) was used to calculate the mean percentages of routine vaccination coverage in different regions of WHO (phase 1).

Microsoft Excel was used to calculate the vaccination coverage with one and two doses of measles vaccines and the vaccination coverage with one, two, and three doses of pertussis vaccines in different countries and to calculate their mean regional values (phase 2).

Microsoft Excel was used to calculate the prevalence of vaccine-induced measles and pertussis protection (phase 3) and to assess the establishment of herd immunity against measles and pertussis (phase 4) in countries of the world and regions of WHO in 2019.

## 3. Results

### 3.1. Mean Percentages of Routine Vaccination Coverage in Regions of WHO in 2019

In 2019, the mean percentages of vaccination coverage in the world ranged from 81.7% for MCV1 to 92.6% for DTP1 vaccine (Table 1). Worldwide, the mean percentages of vaccination coverage were <90% for 10 (76.9%) vaccines and ≥90% for only 3 (23.1%) vaccines: DTP1 vaccine, the last dose of rotavirus vaccine, and the first dose of rubella-containing vaccine (Table 1).

The mean percentages of vaccination coverage were lower than 90% for all routine vaccines in the African region, 12 (92.3%) routine vaccines in the Eastern Mediterranean region, 11 (84.6%) routine vaccines in the Western Pacific region, 8 (61.5%) routine vaccines in the American region, 4 (30%) routine vaccines in the South-East Asia region, and 2 (15.4%) routine vaccines in the European region (Table 1).

In the African region, the mean percentages of vaccination coverage ranged from 65% for MCV1 to 87.5% for DTP1 vaccine (Table 1). In the American Region, the mean percentages of vaccination coverage ranged from 78.2% for the first dose of hepatitis B vaccine to 92.8% for DTP1 vaccine. In the Eastern Mediterranean region, the mean percentages of vaccination coverage ranged from 78.4% for the last dose of rotavirus vaccine to 92% for the first dose of inactivated poliovirus vaccine. In the European region, the mean percentages of vaccination coverage ranged from 69.2% for the last dose of rotavirus vaccine to 97% for DTP1 vaccine. In the South-East Asia region, mean percentages of vaccination coverage ranged from 52.3% for the third dose of pneumococcal conjugate vaccine to 96.2% for DTP1 vaccine. In the Western Pacific region, the mean percentages of vaccination coverage ranged from 78.5% for the third dose of pneumococcal conjugate vaccine to 93.3% for DTP1 vaccine.

In 2019, 31 (16%) countries of the world had a vaccination coverage of ≥95% with MCV2: 2 (4.2%) countries in the African region, 6 (17.1%) countries in the American region, 5 (23.8%) countries in the Eastern Mediterranean region, 8 (15.1%) countries in the European region, 3 (23.7%) countries in the South-East Asia region, and 7 (25.9%) countries in the Western Pacific region. In 2017, 75 (38.7%) countries of the world had a vaccination coverage of ≥95% with MCV1: 7 (14.9%) countries in the African region, 11 (31.4%) countries in the American region, 8 (38.1%) countries in the Eastern Mediterranean region, 6 (54.7%) countries in the European region, 14 (54.5%) countries in the South-East Asia region, and 14 (51.8%) countries in the Western Pacific region.

### 3.2. Routine Vaccination Coverage Variation from 2015 to 2019 in Regions of WHO

In 2015, the mean percentages of vaccination coverage in the world ranged from 69.2% for the first dose of inactivated poliovirus vaccine to 92.9% for DTP1 vaccine (Table 2). In 2015, the mean percentages of vaccination coverage were lower than 90% for 10 (76.9%) routine vaccines (Table 2). The mean vaccination coverage in the world was ≥90% only for DTP1 vaccine, BCG vaccine, and the first dose of rubella-containing vaccine.

The mean percentages of vaccination coverage increased from 2015 to 2019 for four (30.8%) vaccines, decreased for six (46.2%) vaccines, and did not vary for one (7.7%) vaccine (Table 3). Vaccination coverage increases from 2015 to 2019 ranged from 0.3% for the third dose of hepatitis B vaccine to 28% for the first dose of inactivated poliovirus vaccine (Table 3). Vaccination coverage reductions from 2015 to 2019 ranged from −0.1% for MCV1 to −2% for MCV2 (Table 3).

The mean percentages of vaccination coverage increased for the following vaccines: the first dose of hepatitis B vaccine, the third dose of hepatitis B vaccine, the third dose of *Haemophilus influenzae* vaccine, the first dose of inactivated poliovirus vaccine, the third dose of pneumococcal conjugate vaccine, and the last dose of rotavirus vaccination. The mean percentages of vaccination coverage decreased for the following vaccines: BCG vaccine, DTP1 vaccine, MCV1, MCV2, the third dose of poliovirus vaccine, and the first dose of rubella-containing vaccine. The mean vaccination coverage did not vary for DTP3 vaccine from 2015 to 2019.

In the African region, the mean percentages of routine vaccination coverage increased from 2015 to 2019 for six (46.2%) vaccines, decreased for five (38.4%) vaccines, and did not vary for two (15.4%) vaccines (Table 3). Vaccination coverage increases from 2015 to 2019 ranged from 0.1% for BCG vaccine to 140.5% for the first dose of inactivated poliovirus vaccine (Table 3). Vaccination coverage reductions from 2015 to 2019 ranged from −0.1% for MCV1 to −5.4% for the first dose of rubella-containing vaccine (Table 3). The vaccination coverage did not vary from 2015 to 2019 for DTP1 vaccine and MCV2.

In the American region, the mean percentages of routine vaccination coverage increased from 2015 to 2019 for 3 (23.1%) vaccines and decreased for 10 (76.9%) vaccines (Table 3). Vaccination coverage increases from 2015 to 2019 ranged from 0.9% for the third dose of pneumococcal conjugate vaccine to 75.6% for the first dose of inactivated poliovirus vaccine. Vaccination coverage reductions from 2015 to 2019 ranged from −1.8% for MCV1 and the first dose of rubella-containing vaccine to −4.5% for MCV2.

In the Eastern Mediterranean region, the mean percentages of routine vaccination coverage increased from 2015 to 2019 for five (38.5%) vaccines and decreased for eight (61.5%) vaccines (Table 3). Vaccination coverage increases from 2015 to 2019 ranged from 0.2% for DTP1 vaccine and MCV2 to 11.9% for the first dose of inactivated poliovirus vaccine. Vaccination coverage reductions from 2015 to 2019 ranged from −0.2% for five vaccines (DTP3 vaccine, the first dose of hepatitis B vaccine, the third dose of hepatitis B vaccine, the third dose of poliovirus vaccine, and the first dose of rubella-containing vaccine) to −5% for the last dose of rotavirus vaccine.

In the European region, the mean percentages of routine vaccination coverage increased from 2015 to 2019 for 10 (76.9%) vaccines, decreased for 1 (7.7%) vaccine, and did not vary for 2 (15.4%) vaccines (Table 3). Vaccination coverage increases from 2015 to 2019 ranged from 0.4% for the third dose of poliovirus vaccine to 5.5% for the third dose of pneumococcal conjugate vaccine. The vaccination coverage reduction from 2015 to 2019 for the last dose of rotavirus vaccine was −8.3%. The vaccination coverage did not vary from 2015 to 2019 for MCV1 and the first dose of rubella-containing vaccine.

In the South-East Asia region, the mean percentages of routine vaccination coverage increased from 2015 to 2019 for 11 (91.7%) vaccines and decreased for 1 (8.3%) vaccine (Table 3). The vaccination coverage variation was not calculated for the last dose of rotavirus vaccine, because it was not included in national vaccination programs in 2015. Vaccination coverage increases from 2015 to 2019 ranged from 1.2% for BCG and DTP1 vaccines to 97.5% for the third dose of pneumococcal conjugate vaccine. The vaccination coverage reduction for MCV1 was −1.2%.

In the Western Pacific region, the mean percentages of routine vaccination coverage increased from 2015 to 2019 for seven (53.8%) vaccines and decreased for six (46.2%) vaccines (Table 3). Vaccination coverage increases from 2015 to 2019 ranged from 0.2% for BCG vaccine to 41.5% for the last dose of rotavirus vaccine. Vaccination coverage reductions from 2015 to 2019 ranged from −0.1% for DTP1 vaccine to −6% for MCV2.

### 3.3. Mean Percentages of Vaccination Coverage with One and Two Doses of Measles Vaccine and Anti-Measles Herd Immunity Levels in Regions of WHO in 2019

Anti-measles herd immunity levels in 2019 were determined in countries and regions of WHO using the vaccination coverage with one and two doses of measles vaccine in the target vaccination population. The values obtained for different countries of the world (phases 2, 3, and 4 of the analysis) are presented in the Supplementary Table S1.

The mean vaccination coverage with two doses of measles vaccine (indicator of vaccination program performance) was 67.8% in the world, ranging from 38.8% in the African region to 83.9% in the European region (Table 4).

The two-dose measles vaccination coverage was ≥95% in 28 (14.4%) countries of the world (Table 4). The percentage of countries with ≥95% two-dose measles vaccination coverage ranged from 2.1% in the African region to 27.3% in the South-East Asia region (Table 4).

The two-dose measles vaccination coverage was ≥90% in 50 (25.8%) countries of the world. The percentage of countries with two-dose measles vaccination coverage ranged from 2.1% in the African region to 38.1% in the Eastern Mediterranean region (Table 4).

The highest two-dose measles vaccination coverage (98%) was found in the following countries: Seychelles, Cuba, Nicaragua, Saint Vincent and the Grenadines, Bahrain, Morocco, Oman, United Arab Emirates, Iran, Hungary, Turkmenistan, Uzbekistan, Maldives, Sri Lanka, Niue, and Tanga (Table S1).

The mean vaccination coverage with one dose of measles vaccine was 25.8% in the world, ranging from 15.2% in the European region to 44.3% in the African region (Table 4). Children in the target vaccination population had received one dose of measles vaccine in 2019 if they had received MCV1 in 2017 or MCV2 vaccine in 2019.

The mean percentage of children who received 0 doses of measles vaccine (vaccination program performance indicator) was 6.4% in the world, ranging from 0.9% in the European region to 16.9% in the African region (Table 4).

The percentage of countries where all children in the target vaccination population received one or two doses of measles vaccine was 12.9%, ranging from 2.1% in the African region to 28.6% in the Eastern Mediterranean region (Table 4).

Children in the target vaccination population had received two doses of measles vaccine in 2019 if they had received MCV1 in 2017 and MCV2 in 2019. One hundred seventy-six (90.7%) countries included MCV2 in 2019, and all countries included MCV1 in 2017. In 2017, the mean vaccination coverage with MCV1 was 77.7% in the African region, 89.9% in the American region, 86.5% in the Eastern Mediterranean region, 93.1% in the European region, 92.7% in the South-East Asia region, and 87.6% in the Western Pacific region. The mean percentages of MCV1 vaccination coverage were similar in 2017 and 2019.

In 2019, the mean prevalence of individuals with vaccine-induced measles protection in the target measles vaccination population was 88.1%, ranging from 77.6% in the African region to 93.7% in the European region (Table 4). The highest prevalence of individuals with vaccine-induced measles protection (94.9%) was found in the following countries: Seychelles, Cuba, Nicaragua, Saint Vincent and the Grenadines, Bahrain, Morocco, Oman, United Arab Emirates, Iran, Hungary, Turkmenistan, Uzbekistan, Maldives, Sri Lanka, Niue, and Tanga (Table S1).

Based on the global mean levels of measles protection, anti-measles herd immunity was not established against measles viruses with R_o_ ≥ 10 in the target vaccination population, because the global mean prevalence of individuals with vaccine-induced measles protection (88.1%) was lower than the critical prevalence of 90% required to establish herd immunity against measles viruses with an R_o_ value of 10 (Table 4). In the world, the prevalence of individuals in the target vaccination population with vaccine-induced measles protection is high, but it is not sufficient to block transmission of measles viruses with R_o_ ≥ 10.

Based on the regional mean levels of measles protection, anti-measles herd immunity was established in the European region against measles viruses with R_o_ ≤ 15 but not against measles viruses with R_o_ ≥ 16, because the mean prevalence of vaccine-induced measles protection (93.7%) was higher than the critical prevalence required to establish herd immunity against measles viruses with R_o_ = 15 (93.3%) and it was lower than the critical prevalence required against viruses with R_o_ = 16 (93.7%) (Table 4). In the South-East Asia region, anti-measles herd immunity was established against measles viruses with R_o_ ≤ 15 but not against measles viruses with R_o_ ≥ 16, because the mean prevalence of vaccine-induced measles protection (93.2%) was higher than the critical prevalence for measles viruses with R_o_ = 15 (93.3%) and it was lower than the critical prevalence required for measles viruses with R_o_ = 16 (93.7%). In the American region, anti-measles herd immunity was established against measles viruses with R_o_ ≤ 12 but not against measles viruses with R_o_ ≥ 13, because the mean prevalence of vaccine-induced measles protection (91.8%) was higher than the critical prevalence for measles viruses with R_o_ = 12 (91.7) and it was lower than the critical prevalence required for measles viruses with R_o_ = 13 (93.2%). In the African and Western Pacific regions, anti-measles herd immunity could not be established against measles viruses with R_o_ ≥ 10, because in these regions the mean prevalence of vaccine-induced measles protection was lower than the critical prevalence required to establish herd immunity against measles viruses with R_o_ = 10 (90%).

Based on country-specific measles protection levels, anti-measles herd immunity was established in 137 (70.6%) countries against measles viruses with R_o_ ≤ 10, in 100 (51.5%) countries against measles viruses with R_o_ ≤ 15, in 63 (32.5%) countries against measles viruses with R_o_ ≤ 18, and in 31 (16%) countries against measles viruses with R_o_ ≤ 19, and it was not established in any country against measles viruses with R_o_ ≥ 20 (Table 4 and Table S2). The European region had the highest percentage of countries, and the African region had the lowest percentage of countries with herd immunity established against different measles viruses. In the European region, herd immunity was established in 50 (94.3%) countries against measles viruses with R_o_ ≤ 10, in 43 (81.1%) countries against measles viruses with R_o_ ≤ 15, in 22 (41.5%) countries against measles viruses with R_o_ ≤ 18, and in 9 (17%) countries against measles viruses with R_o_ ≤ 19 (Table 4). In the African region, herd immunity was established in 20 (40.4%) countries against measles viruses with R_o_ ≤ 10, in 7 (14.9%) countries against measles viruses with R_o_ ≤ 15, and in 1 (2.2%) country against measles viruses with R_o_ ≤ 19 (Table 4).

### 3.4. Mean Percentages of Vaccination Coverage with One, Two, and Three Doses of Pertussis Vaccines and Anti-Pertussis Herd Immunity Levels in Regions of WHO in 2019

Anti-pertussis herd immunity levels in the target vaccination population (children aged year) in 2019 were determined in countries and regions of WHO using the vaccination coverage with one, two, and three doses of pertussis vaccine in 2019. The values obtained for different countries of the world (phases 2, 3, and 4 of the analysis) are presented in the Supplementary Table S2.

The global mean vaccination coverage with one, two, and three doses of DTP vaccine were 4.3%, 18%, and 77%, respectively (Table 5). The mean vaccination coverage with three doses of DTP vaccine (indicator of vaccination program performance) ranged from 64.1% in the African region to 88.1% in the European region (Table 5).

The vaccination coverage with three doses of pertussis vaccine was ≥95% in 35 (18%) countries of the world (Table 5). The percentage of countries with ≥95% three-dose pertussis vaccination coverage ranged from 4.2% in the African region to 33.3% in the Western Pacific region (Table 5).

The vaccination coverage with three doses of pertussis vaccine was ≥90% in 66 (34%) countries of the world (Table 5). The percentage of countries with ≥90% three-dose pertussis vaccination coverage ranged from 6.4% in the African region to 54.5% in the South-East Asia region (Table 5).

The highest vaccination coverage with three doses of pertussis vaccine (97%) was found in the following countries: Seychelles, Cuba, Dominica, Guyana, Bahrain, Morocco, Oman, United Arab Emirates, Iran, Albania, Andorra, Greece, Hungary, Latvia, Luxemburg, Monaco, Portugal, Turkey, Turkmenistan, Maldives, Sri Lanka, Brunei, China, Fiji, Niue, and Tanga (Table S2).

The mean vaccination coverage with two doses of DTP vaccine ranged from 11.1% in the European region to 26.1% in the African region. The mean vaccination coverage with one dose of DTP vaccine ranged from 0.8% in the European region to 8.3% in the African region (Table 5).

The mean percentage of children that received 0 doses of DTP vaccine (indicator of vaccination program performance) was 0.7% in the world, ranging from 0% in the European and South-East Asia regions to 1.5% in the African region (Table 5).

The percentage of countries where all children in the target vaccination population (one year old) had received at least one dose (one, two, or three doses) of pertussis vaccine in 2019 was 64.4%, ranging from 42.5% in the African region to 89.4% in the European region (Table 5).

The mean prevalence of individuals with vaccine-induced pertussis immunity in the target pertussis vaccination population was 81.1% in 2019, ranging from 78.9% in the African region to 83% in the European region (Table 5). The highest pertussis immunity (83.8%) was found in the following countries: Seychelles, Cuba, Cuba, Dominica, Guyana, Bahrain, Morocco, Oman, United Arab Emirates, Iran, Albania, Andorra, Greece, Hungary, Latvia, Luxemburg, Monaco, Portugal, Turkey, Turkmenistan, Maldives, Sri Lanka, Brunei, China, Fiji, Niue, and Tanga (Supplementary Table S2).

Based on the global mean levels of pertussis protection, anti-pertussis herd immunity was not established in the world in the target vaccination population against pertussis agents with R_o_ ≥ 10, because the global mean prevalence of individuals with vaccine-induced measles protection (81.1%) was lower than the critical prevalence of 90% required to establish herd immunity against measles viruses with an R_o_ value of 10 (Table 5).

Based on the regional mean levels of pertussis protection, anti-pertussis herd immunity was not established in the six regions, because the mean prevalence of vaccine-induced measles protection was lower than the critical prevalence of 90% required to establish herd immunity against measles viruses with an R_o_ value of 10 (Table 5).

Based on country-specific pertussis protection levels, anti-pertussis herd immunity was not established in the six regions, because the mean prevalence of vaccine-induced measles protection was lower than the critical prevalence of 90% required to establish herd immunity against measles viruses with an R_o_ value of 10 (Table 5 and Supplementary Table S2).

## 4. Discussion

The study found that the mean percentages of vaccination coverage in the world in 2019 were lower than the objective of 90% for 77% of routine vaccines and that percentages of vaccination coverage decreased for 46.2% of routine vaccines from 2015 to 2019. The results obtained in this study could explain the persistence of infections that can be prevented by routine vaccinations in different regions of WHO. In 2019, the mean percentages of vaccination coverage in in the world were >90% only for DTP1 vaccine, the last dose of rotavirus vaccine, and the first dose of rubella-containing vaccine and were ≤90% for BCG vaccine, DTP3 vaccine, the first and third doses of hepatitis B vaccine, MCV1 and MCV2, the first dose of inactivated poliovirus vaccine, and the third dose of poliovirus vaccine.

The worst situation was found in the African, Eastern Mediterranean, and Western Pacific regions, where the mean percentages of vaccination coverage were lower than 90% for more than 90% of routine vaccines. Meanwhile, in the European, South-East Asia, and American regions, the mean percentages of vaccination coverage were lower than 90% for 15.4%, 30%, and 61.5% of routine vaccines, respectively.

The analysis of the vaccination coverage variation from 2015 to 2019 carried out in this study showed that routine vaccinations did not improve for BCG vaccine, DTP1 vaccine, MCV1, MCV2, the third dose of poliovirus vaccine, the first dose of rubella-containing vaccine, and DTP3 vaccine. These vaccines represent 54% of routine vaccines. The worst variation was found in the American and Eastern Mediterranean regions, where the mean percentages of vaccination coverage decreased for more than 50% of routine vaccines since 2015. On the contrary, in the European and Western Pacific regions, the mean percentages of vaccination coverage increased for more than 90% of routine vaccines since 2015.

The study found low mean percentages of vaccination coverage with two doses of measles vaccine (67.8%) in the world in 2019. This low figure can be attributed to a very low mean coverage in the African region (38.8%) and the <90 vaccination coverage in other regions. In fact, 74.2% and 85.6% of the countries of the world had <90% and <95% vaccination coverage with two doses of measles vaccine, respectively.

The study found low mean percentages of vaccination coverage with three doses of DTP vaccine (77%) in the world in 2019. The low figure can be attributed also to a very low coverage in the African region (64.1%) and the <90% vaccination coverage in other regions. In fact, 66% and 82% of the countries of the world had <90% and <95% vaccination coverage with three doses of DTP vaccine, respectively.

The <90% vaccination coverage found in this study for most routine vaccines worldwide and in the African American, Eastern Mediterranean, and Western Pacific regions can be explained by the following factors: insufficient resources for national vaccination programs, insufficient access to vaccinations, vaccination hesitance and lack of vaccine confidence, and anti-vaccination activities. Anti-vaccinationists can reduce the percentages of routine vaccination coverage in countries, areas, and communities by questioning the effectiveness, safety, and necessity for routine vaccinations [33]. Nevertheless, many studies have demonstrated that routine vaccinations are effective, safe, and cost-effective interventions [2,3]. Vaccines have saved countless lives, lowered the global incidence of polio by 99%, and reduced illness, disability, and death from diphtheria, tetanus, whooping cough, measles, *Haemophilus influenzae* type b disease, and epidemic meningococcal A meningitis [2].

In the European and South-East Asia regions, 84.6% and 69.2% of routine vaccines, respectively, had >90% mean vaccination overage, which was higher than in other regions. In addition, the mean percentages of vaccination coverage improved from 2015 to 2019 for 92.3% of routine vaccines in the European region and for 91.7% of routine vaccines in the South-East Asia regions. Nevertheless, the mean vaccination coverage with two doses of measles vaccine and three doses of pertussis vaccine in these regions was lower than 85%.

The results obtained in this study show that the persistence of measles in the world in 2019 could be explained by low percentages of two-dose measles vaccination coverage and low herd immunity levels in the target measles vaccination population in all regions of WHO. First, the percentages of vaccination coverage with two doses of measles vaccine were lower than 90% in all regions of the world: 38.8% in the African region; 71–73% in the Americas, Eastern Mediterranean, and Western Pacific regions; and 81–83% in the European and South-East Asia regions. Second, the mean vaccination coverage with MCV1 and MCV2 in the world decreased from 2015 to 2019 by 0.1% and 2%, respectively. Third, worldwide, the percentage of countries with ≥95% two-dose measles vaccination coverage was only 14.4%, (2.1% in the African region and 14–27% in other regions of WHO). Fourth, anti-measles herd immunity was established in the target vaccination population against measles viruses with R_o_ values of 18 in 32.5% of the countries of the world. However, herd immunity was not established against measles viruses with R_o_ ≥ 10 in the African and Eastern Mediterranean regions, measles viruses with R_o_ ≥ 11 in the Western Pacific region, and measles virus with R_o_ ≥ 13 in the American, European, and South-East Asia regions.

A study carried out in 2019 found a significant negative correlation between the incidence of measles in 2017–2018 and two-dose measles vaccination and herd immunity levels in the target measles vaccination population in countries of the European Union during 2015–2017 [23]. This correlation indicates that vaccination of children with two doses of measles vaccine can reduce the incidence of measles in children and in other population groups [23]. Measles vaccination with the two-dose measles vaccine can reduce measles incidence by direct and indirect protection in the target vaccination population and by indirect herd immunity protection in other population groups. Another study found that to meet the goal of measles elimination in Europe, it is necessary to achieve percentages of two-dose measles vaccination coverage of at least 97% [20].

Sustained high vaccination coverage with two doses of measles vaccines is the key preventive measure to achieve measles control and measles elimination [2,9,20]. All WHO regions have proposed to achieve measles elimination [8]. Measles vaccination reduced the measles incidence from 2000 to 2016, but measles resurgence after 2016 marked a significant step backward in the progress toward global measles elimination [26,34]. Reported measles cases increased by 556% and the global measles mortality increased by 50% from 2016 to 2019 [5]. The results obtained in this study indicate that vaccination coverage with two doses of measles vaccine and anti-measles herd immunity levels should be increased in order to achieve measles control and measles elimination in the world. While advanced immunization information systems could be developed for detecting unvaccinated individuals and areas and population groups with low routine vaccination rates, they cannot detect areas and population groups with low immunity levels due to primary vaccination failures and waning vaccine-induced immunity [35]. For this reason, new immunization strategies should be developed to detect populations and areas with gaps in measles immunity, where susceptible individuals should be identified and immunized [20,23,26,34].

Sustained high vaccination coverage with three doses of pertussis vaccines in children aged one year is the key preventive measure to achieve pertussis control [2,8,36]. The results obtained in this study show that the persistence of pertussis in the world in 2019 could be explained by low percentages of vaccination coverage with three doses of DTP vaccine, low effectiveness of pertussis vaccines, and low herd immunity levels in the target measles vaccination population in all regions of WHO. First, the percentages of vaccination coverage with three doses of DTP vaccine were lower than 90% in all regions of the world: 64.1% in the African region; 71–79% in the American, Eastern Mediterranean, and Western Pacific regions; and 85–88% in the European and South-East Asia regions. Second, the percentages of countries where all children in the target vaccination population had received at least one dose of DTP vaccine in 2019 was lower than 90% in all regions: 42–60% in the American, Eastern Mediterranean, and Western Pacific regions; 72–78% in the South-East Asia and Western Pacific regions; and 89% in the European region. Third, the percentage of countries with ≥95% vaccination coverage with three doses of DTP vaccine was lower than 30% in all regions of WHO and only 4.2% in the African region. Fourth, worldwide, the mean percentage of vaccination coverage with DTP1 vaccine decreased by 0.3% from 2015 to 2019. In addition, 18 countries of the world experienced decreases of more than 10% in DTP3 vaccine coverage from 2010 to 2019 [36]. Fifth, the mean prevalence of individuals with anti-pertussis vaccine-induced immunity in the target vaccination population was not sufficient to block the transmission of pertussis agents with R_o_ ≥ 10 in all regions of WHO.

Indicators of vaccination program performance based on the vaccination coverage with DTP1 and DTP3 vaccines have been considered in other studies [19,36]. Chard et al. [19] defined the percentage of zero-dose children in 2019 as the percentage of children (aged one year) who had not received DTP1 vaccine, and defined the percentage of children who completed the three-dose DTP series in 2019 as the percentage of children (aged one year) who had received DTP3 vaccine. On the basis of these indicators, Chard et al. determined that 13.8 million children worldwide were zero-dose children and that 19.7 million children did not complete the three-dose DTP series in 2019 [19]. These numbers were obtained taking into account aggregated percentages of vaccination coverage of 90% for DTP1 vaccine and 85% for DTP3 vaccine [19].The aggregated percentages of DTP1 and DTP3 vaccination coverage in 2019 were slightly lower than the mean percentages of vaccination coverage obtained in the present study (93.2% for DTP1 vaccine and 88.7% for DTP3 vaccine), because the aggregated vaccination coverage takes into account the size of the target vaccination populations in different countries. Nevertheless, based on the results obtained in this study, the number of children who completed the three-dose DTP series must be lower than 19.7 million because the global mean vaccination coverage with three doses of DTP vaccine obtained in this study (77%) is lower than the three-dose DTP vaccination coverage assumed in the study of Chard et al. (88.7%) [19]. The results obtained in this study reveal that the number of zero-dose children worldwide must be lower than 13.8 million because the mean zero-dose vaccination coverage obtained in this study (0.7%) is lower than the zero-dose vaccination coverage assumed by Chard et al. (6.8%) [19].

This study had several limitations. First, herd immunity was assessed by comparing the prevalence of individuals with vaccine-induced protection and the critical prevalence associated with herd immunity for measles and pertussis. This method is based on the following assumptions: (1) homogeneous mixing of individuals within the population and (2) homogeneous distribution of protected individuals within the population [30,37]. Nevertheless, it is possible to assume a homogeneous mixing of persons and ahomogeneous distribution of protected individuals within the measles and pertussis target vaccination population [26,32]. Second, anti-measles and anti-pertussis herd immunity was assessed in different regions of WHO using the critical prevalence of protected individuals for measles viruses with R_o_ values from 10 to ≥20 for measles and from 10 to ≥18 for pertussis. Values of R_o_ lower than 10 would increase the percentage of countries with herd immunity. Nevertheless, the range of R_o_ values assumed in this study have been found in most studies assessing R_o_ values for measles and pertussis [30,31]. Third, the prevalence of individuals with vaccine-induced measles protection in different countries was calculated by assuming measles vaccination effectiveness of 95% in preventing measles cases with two doses of vaccine and 92% with one dose of vaccine. Higher and lower values of effectiveness would increase and decrease the percentage of countries with herd immunity, respectively. Nevertheless, these values of effectiveness were obtained from the Cochrane Collaboration review and evaluative studies [24]. Fourth, the prevalence of individuals in different countries with vaccine-induced pertussis protection was calculated by assuming pertussis vaccination effectiveness of 84%, 77%, and 59% in preventing pertussis cases with one, two, and three doses of pertussis vaccine, respectively. Higher and lower values of effectiveness would increase and decrease the percentage of countries with herd immunity, respectively. Nevertheless, these values of effectiveness were obtained from the Cochrane Collaboration review and evaluative studies [27]. Five, the analysis carried out in this study used the information about routine vaccination of the WHO–UNICEF global and regional immunization information system. This information is subject to potential bias due to misreporting of the percentages of routine vaccination coverage. Nevertheless, the information reported by the WHO–UNICEF global and regional immunization information system is validated periodically by the World Health Organization, and there are no alternative consistent sources of information.

The annual percentages of routine vaccination coverage reported by different countries to the WHO–UNICEF global and regional immunization information system are obtained using two basic methods: administrative data and survey data [18]. The annual vaccination coverage determined using administrative data is obtained from the number of vaccines administered in different vaccination centers (numerator) and the target population (denominator). The main advantage of this information system is that it is possible to collect and review the number of vaccines administered per month in different vaccination centers and obtain the aggregated national vaccination coverage per year. The main disadvantage is that it is subject to numerator and denominator bias. The vaccination coverage obtained from national representative surveys is subject to less potential bias than the administrative method. The main disadvantages of the survey method are that it is necessary to obtain a representative sample of the population (numerator and denominator) and it is subject to potential respondent recall bias.

Significant elimination efforts have been made since 2011 to develop the Global Vaccine Action Plan. However, the results obtained in this study indicate the necessity of increasing the percentages of routine vaccination coverage. This goal can be realized by giving more resources to vaccination programs, implementing advanced vaccination programs, and developing activities to reduce vaccine hesitancy and increase vaccine confidence. The recommendations to improve the Global Vaccine Action Plan include developing a more country-focused approach, providing technical assistance according to specific needs, and considering immunization programs as one part of integrated disease control programs [38,39]. The Global Routine Immunization Strategies and Practices document of WHO [11] proposes the following transformative investments to reassert immunization as the foundation of sustained decreases in morbidity and mortality from vaccine-preventable diseases: (1) invest in a national team to expertly manage national vaccination programs, (2) invest in strategies to identify and immunize undervaccinated and unvaccinated persons, (3) invest in vaccinators and district managers, (4) invest in modernizing vaccine supply chains and management, and (5) invest in information systems that identify and tracks each person’s immunization status.

## 5. Conclusions

The mean percentages of routine vaccination coverage were lower than 90% for 77% of routine vaccines in 2019 and decreased from 2015 to 2019 for 46% of routine vaccines. The percentages of vaccination coverage with two doses of measles vaccine and three doses of pertussis were low in all regions of WHO. Therefore, it is necessary to improve routine immunization programs in order to achieve at least 90% vaccination coverage for all routine vaccines in all countries. Moreover, ≥95% vaccination coverage with two doses of measles vaccine and three doses of DTP vaccine must be reached in all countries to achieve measles control and elimination and pertussis control, respectively.

## Figures and Tables

**Table 1:** Mean vaccination coverage for different routine vaccines in different regions of the World Health Organization in 2019.

Vaccine	World		African Region		American Region		Eastern Mediterranean Region		European Region		South-East Asia Region		Western Pacific Region	
	%	*n*	%	*n*	%	*n*	%	*n*	%	*n*	%	*n*	%	*n*
BCG	89.9	155	87.4	47	90.7	27	85.6	19	92.8	27	95.3	11	91.8	24
DTP1	92.6	194	87.5	47	92.8	35	89.8	21	97	53	96.2	11	93.3	27
DTP3	88.2	194	81.1	47	88.3	35	85	21	94.4	53	93.5	11	88.8	27
HepB1	83.7	95	79.3	7	78.2	22	80.6	14	95.1	21	76.1	8	84.6	23
HepB3	87.5	189	81	47	87.4	35	84.8	21	92.8	48	93.5	11	89.1	27
Hib3	87.8	191	81	47	88.3	35	85	21	93.7	52	93.2	10	87.3	26
IPV1	88.7	193	78.6	47	91.9	35	92	21	96.2	52	90.4	11	88	27
MCV1	87.6	194	78.3	47	90.3	35	85	21	93	53	93.6	11	89.1	27
MCV2	81.7	176	65	32	80.7	35	82.4	20	91.6	52	87.4	11	80.7	26
PCV3	83.3	142	81.6	40	86.3	24	84.1	15	89.6	40	52.3	6	78.5	17
Pol3	83.3	194	80.4	47	88.9	35	85.6	21	94.4	53	94.1	11	88.9	27
RCV1	90.1	170	85.1	29	90.3	35	88.8	16	93	53	93.2	10	89	27
Rota	92.3	99	84.1	35	81.6	20	78.4	14	69.2	21	53	1	82.3	8
Vaccines with <90% coverage	76.9		100		61.5		92.3		15.4		30.8		94.6	

*n*: number of countries with vaccine in national vaccination coverage; BCG: Bacillus Calmette–Guérin vaccine against tuberculosis; DTP1: first dose of diphtheria-tetanus-pertussis vaccine; DTP3: third dose of diphtheria-tetanus-pertussis vaccine; HepB1: first dose of hepatitis B vaccine; HepB3: third dose of hepatitis B vaccine; Hib3: third dose of Haemophilus influenzae vaccine; IPV1: first dose of inactivated poliovirus vaccine; MCV1: first dose of measles-containing vaccine; MCV2: second dose of measles-containing vaccine; PCV3: third dose of pneumococcal conjugate vaccine; Pol3: third dose of polio vaccine; RCV1: first dose of rubella-containing vaccine; Rota: last dose of rotavirus vaccine.

**Table 2:** Mean vaccination coverage for different routine vaccines in different regions of the World Health Organization in 2015.

Vaccine	World		African Region		American Region		Eastern Mediterranean Region		European Region		South-East Asia Region		Western Pacific Region	
	%	*n*	%	*n*	%	*n*	%	*n*	%	*n*	%	*n*	%	*n*
BCG	90.2	157	87.3	47	95.5	27	87.5	19	89.1	29	94.2	11	91.6	24
DTP1	92.9	194	87.6	47	95.6	47	89.7	21	96.5	53	94.5	11	93.4	27
DTP3	88.2	194	80.1	47	90.7	47	85.1	21	93.7	53	92.5	11	89.1	27
HepB1	81.1	77	83.8	3	72.6	15	79.9	13	94.0	22	72.1	3	80.5	21
HepB3	87.2	185	79.8	47	89.7	35	85.0	21	91.6	45	92.5	11	89.3	26
Hib3	87.1	191	79.7	47	90.3	35	85.5	21	91.7	52	87.6	10	88.8	26
IPV1	69.2	129	32.6	20	52.2	24	77.7	15	93.6	43	46.4	8	77.2	19
MCV1	87.7	194	78.3	47	92.0	35	84.8	21	93.0	53	91.1	11	88.9	27
MCV2	83.3	155	65.0	23	84.6	29	81.1	20	90.7	51	82.2	9	85.9	23
PCV3	79.3	121	73.4	36	85.5	24	87.8	13	84.9	30	26.5	2	72.5	16
Pol3	88.5	194	80.0	47	90.9	47	85.8	21	94.0	53	92.5	11	89.8	27
RCV1	91.4	14	90.0	8	92.0	35	89.0	16	93.0	53	94.3	7	88.5	27
Rota	76.3	78	73.5	28	84.5	18	82.5	11	75.5	14	−	0	58.1	7
Vaccines with <90% coverage	76.9		92.3		46.1		100		23.1		33.3		94.6	

*n*: number of countries with vaccine in national vaccination coverage; BCG: Bacillus Calmette–Guérin vaccine against tuberculosis; DTP1: first dose of diphtheria-tetanus-pertussis vaccine; DTP3: third dose of diphtheria-tetanus-pertussis vaccine; HepB1: first dose of hepatitis B vaccine; HepB3: third dose of hepatitis B vaccine; Hib3: third dose of Haemophilus influenzae vaccine; IPV1: first dose of inactivated poliovirus vaccine; MCV1: first dose of measles-containing vaccine; MCV2: second dose of measles-containing vaccine; PCV3: third dose of pneumococcal conjugate vaccine; Pol3: third dose of polio vaccine; RCV1: first dose of rubella-containing vaccine; Rota: last dose of rotavirus vaccine.

**Table 3:** Variation (%) from 2015 to 2019 for the mean percentages of routine vaccination coverage in different regions of the World Health Organization.

Vaccine	World	African Region	American Region	Eastern Mediterranean Region	European Region	South-East Asia Region	Western Pacific Region
BCG	−0.3	0.1	−5.0	−2.1	4.2	1.2	0.2
DTP1	−0.3	0	−3.0	0.2	0.6	1.7	−0.1
DTP3	0	1.2	−2.7	−0.2	0.8	1.2	−0.3
HepB1	3.2	−5.3	7.7	0.8	1.2	5.5	5.0
HepB3	0.3	1.6	−2.5	−0.2	1.3	1.2	−0.3
Hib3	0.9	1.6	−2.2	−0.2	2.2	6.4	−0.7
IPV1	28	140.5	75.6	11.9	3.3	94.9	14
MCV1	−0.1	−0.1	−1.8	0.2	0	2.8	0.3
MCV2	−2.0	0	−4.5	1.6	0.9	6.3	−6.0
PCV3	5	11.1	0.9	−4.3	5.5	97.5	8.3
Pol3	−0.2	0.5	−2.2	−0.2	0.4	1.8	−1.0
RCV1	−1.4	−5.4	−1.8	−0.2	0	−1.2	0.5
Rota	3.8	14.5	−3.5	−5.0	−8.3	−	41.5

BCG: Bacillus Calmette–Guérin vaccine against tuberculosis; DTP1: first dose of diphtheria-tetanus-pertussis vaccine; DTP3: third dose of diphtheria-tetanus-pertussis vaccine; HepB1: first dose of hepatitis B vaccine; HepB3: third dose of hepatitis B vaccine; Hib3: third dose of Haemophilus influenzae vaccine; IPV1: first dose of inactivated poliovirus vaccine; MCV1: first dose of measles-containing vaccine; MCV2: second dose of measles-containing vaccine; PCV3: third dose of pneumococcal conjugate vaccine; Pol3: third dose of polio vaccine; RCV1: first dose of rubella-containing vaccine; Rota: last dose of rotavirus vaccine.

**Table 4:** Mean vaccination coverage with zero, one, and two doses of measles vaccines, mean prevalence of individuals in the target vaccination population with vaccine-induced measles protection, percentage of countries with anti-measles herd immunity established in the target population vaccination, and percentage of countries with other measles vaccination indicators in regions of the World Health Organization (WHO) in 2019.

	World	African Region	American Region	Eastern Mediterranean Region	European Region	South-East Asia Region	Western Pacific Region
No. of countries	194	47	35	21	53	11	27
Mean vaccination coverage (%) with zero, one, and two doses of measles vaccine							
2 doses	67.8	38.8	73.2	71.4	83.9	81.5	71.2
1 dose	25.8	44.3	24.1	22.1	15.2	17.1	23.1
0 doses	6.4	16.9	2.7	6.5	0.9	1.4	5.7
Mean prevalence (%) of individuals in the target vaccination population with vaccine-induced measles immunity							
Measles immunity	88.1	77.6	91.8	88.2	93.7	93.2	88.8
Percentage of countries with vaccination coverage of ≥95% and ≥90% with two doses of measles vaccine							
≥95%	14.4	2.1	14.3	23.8	15.1	27.3	22.2
≥90%	25.8	2.1	22.9	38.1	35.8	36.4	37.0
Percentage of countries where all children received one or two doses of measles vaccine							
	12.9	2.1	14.3	28.6	9.4	27.3	18.5
Percentage of countries with herd immunity against measles viruses with R_o_ from 10 to ≥20							
R_o_ = 10	70.6	40.4	74.3	66.7	94.3	81.8	70.4
R_o_ = 12	63.9	29.8	65.7	61.9	90.6	72.7	66.7
R_o_ = 15	51.5	14.9	45.7	52.4	81.1	54.5	63.0
R_o_ = 18	32.5	8.5	22.9	38.1	41.5	54.5	40.7
R_o_ = 19	16.0	2.2	17.1	28.6	17.0	27.3	22.2
R_o_ ≥ 20	0	0	0	0	0	0	0

**Table 5:** Mean vaccination coverage with zero, one, two, and three doses of pertussis vaccine, mean prevalence of individuals in the target vaccination population with vaccine-induced pertussis protection, percentage of countries with anti-pertussis herd immunity established in the target vaccination population, and other pertussis vaccination indicators in regions of WHO in 2019.

	World	African Region	American Region	Eastern Mediterranean Region	European Region	South-East Asia Region	Western Pacific Region
No. of countries	194	47	35	21	53	11	27
Mean vaccination coverage (%) with zero, one, two, and three doses of DTP vaccine							
3 doses	77.0	64.1	75.9	71.3	88.1	85.9	79.8
2 doses	18.0	26.1	20.1	20.9	11.1	12.9	14.7
1 dose	4.3	8.3	3.7	6.6	0.8	1.2	4.3
0 doses	0.7	1.5	0.3	1.2	0.0	0.0	1.2
Mean prevalence (%) of individuals in the target vaccination population with vaccine-induced pertussis immunity							
Pertussis immunity	88.1	77.6	91.8	88.2	93.7	93.2	88.8
Percentage of countries with vaccination coverage of ≥95% and ≥90% with three doses of DTP vaccine							
≥95%	18.0	4.2	14.3	23.8	22.6	18.2	33.3
≥90%	34.0	6.4	25.7	28.6	52.8	54.5	51.8
Percentage of countries where all children received one, two, or three doses of DTP vaccine							
	64.4	42.5	57.1	52.4	89.4	72.7	77.8
Percentage of countries with herd immunity established against pertussis viruses with R_o_ from 10 to ≥18							
R_o_ = 10	0	0	0	0	0	0	0
R_o_ = 12	0	0	0	0	0	0	0
R_o_ = 15	0	0	0	0	0	0	0
R_o_ ≥ 18	0	0	0	0	0	0	0

## Data Availability

The results obtained in this study were obtained using the information about WHO on WHO-UNICEF Estimates of Routine Vaccines: https://apps.who.int/immunization_monitoring/globalsummary/timeseries/tswucoveragexxx.html (accessed on 15 January 2021).

## Supplementary Materials

The following are available online at https://www.mdpi.com/2076-393X/9/3/256/s1: Table S1: Measles vaccination coverage and measles protection in countries of the world in 2019: (1) vaccination coverage with the second dose of measles-containing vaccine (MCV2) in 2019 and the first dose of measles-containing vaccine (MCV1) in 2017, (2) vaccination coverage with one and two doses of measles vaccine in the target vaccination population, (3) measles protection in terms of prevalence of individuals with vaccine-induced measles protection in the target vaccination population, and (4) vaccination coverage with one or two doses of measles vaccine; Table S2: Pertussis vaccination coverage and pertussis protection in countries of the world in 2019: (1) vaccination coverage with the first, second, and third doses of diphtheria-tetanus-pertussis vaccine (DTP1, DTP2, and DTP3 vaccines, respectively); (2) vaccination coverage with one, two, and three doses of DTP vaccine in the target vaccination population; (3) pertussis protection in terms of prevalence of individuals with vaccine-induced pertussis protection in the target vaccination population; and (4) vaccination coverage with one, two, or three doses of DTP vaccine.
