# Supplementary material for: Vaccination Coverage for Routine Vaccines and Herd Immunity Levels against Measles and Pertussis in the World in 2019
Source: Vaccines (Basel). 2021 Mar 13;9(3):256. doi: 10.3390/vaccines9030256 (PMC7999208; doi:10.3390/vaccines9030256)
Supplement: Supplementary file 1 [file vaccines-09-00256-s001.pdf]

Table S1: Measles vaccination coverage and measles protection in countries of the world in 2019: 1) Vaccination coverage with the second dose of measles containing vaccine (MCV2) in 2019 and the first dose of measles containing vaccine (MCV1) in 2017; 2) vaccination coverage with one and two doses of measles vaccine in the target vaccination population; 3) measles protection in terms of prevalence of individuals with vaccine-induced measles protection in the target vaccination population; and 4) vaccination coverage with one or two doses of measles vaccine

Region	Country name	Vaccination coverage (%)		Vaccination coverage (%)		Measles Protection (%)	Vaccination coverage (%) with one or two doses of measles vaccine
		MCV2 vaccine (2019)	MCV1 vaccine (2017)	2 doses	1 dose		
AFR	Seychelles	99.0	99.0	98.0	2.0	94.9	100.0
AFR	Mauritius	99.0	89.0	88.1	11.8	94.5	99.9
AFR	Eritrea	88.0	99.0	87.1	12.8	94.5	99.9
AFR	Rwanda	92.0	97.0	89.2	10.5	94.5	99.8
AFR	Cabo Verde	91.0	96.0	87.4	12.3	94.3	99.6
AFR	Ghana	83.0	95.0	78.9	20.3	93.6	99.2
AFR	Botswana	76.0	97.0	73.7	25.6	93.5	99.3
AFR	Zambia	66.0	96.0	63.4	35.3	92.6	98.6
AFR	Lesotho	82.0	90.0	73.8	24.4	92.6	98.2
AFR	Sao Tome and Principe	81.0	90.0	72.9	25.2	92.4	98.1
AFR	Mozambique	85.0	87.0	74.0	24.1	92.4	98.1
AFR	Burundi	80.0	90.0	72.0	26.0	92.3	98.0
AFR	Senegal	78.0	90.0	70.2	27.6	92.1	97.8
AFR	Zimbabwe	75.0	90.0	67.5	30.0	91.7	97.5
AFR	Algeria	77.0	88.0	67.8	29.5	91.5	97.2
AFR	Eswatini	75.0	89.0	66.8	30.5	91.5	97.3
AFR	United Republic of Tanzania	72.0	90.0	64.8	32.4	91.4	97.2
AFR	Burkina Faso	71.0	88.0	62.5	34.0	90.7	96.5
AFR	Gambia	61.0	90.0	54.9	41.2	90.1	96.1
AFR	Malawi	75.0	83.0	62.3	33.5	90.0	95.8
AFR	Sierra Leone	72.0	80.0	57.6	36.8	88.6	94.4
AFR	Kenya	45.0	89.0	40.1	53.9	87.6	94.0
AFR	Togo	67.0	77.0	51.6	40.8	86.6	92.4
AFR	Niger	58.0	82.0	47.6	44.9	86.5	92.4
AFR	Namibia	56.0	80.0	44.8	46.4	85.2	91.2
AFR	Comoros		90.0	0.0	90.0	82.8	90.0
AFR	Liberia	13.0	87.0	11.3	77.4	81.9	88.7
AFR	South Africa	54.0	70.0	37.8	48.4	80.4	86.2
AFR	Guinea-Bissau		86.0	0.0	86.0	79.1	86.0
AFR	Uganda		83.0	0.0	83.0	76.4	83.0
AFR	Mauritania		78.0	0.0	78.0	71.8	78.0

AFR	Ethiopia	41.0	59.0	24.2	51.6	70.5	75.8
AFR	Congo	9.0	70.0	6.3	66.4	67.1	72.7
AFR	Mali	4.0	70.0	2.8	68.4	65.6	71.2
AFR	Benin		70.0	0.0	70.0	64.4	70.0
AFR	Côte d'Ivoire		70.0	0.0	70.0	64.4	70.0
AFR	Angola	45.0	42.0	18.9	49.2	63.2	68.1
AFR	Cameroon		65.0	0.0	65.0	59.8	65.0
AFR	Gabon		63.0	0.0	63.0	58.0	63.0
AFR	Madagascar		60.0	0.0	60.0	55.2	60.0
AFR	Nigeria	9.0	54.0	4.9	53.3	53.6	58.1
AFR	Democratic Republic of the Congo		57.0	0.0	57.0	52.4	57.0
AFR	Equatorial Guinea		53.0	0.0	53.0	48.8	53.0
AFR	South Sudan		50.0	0.0	50.0	46.0	50.0
AFR	Central African Republic		49.0	0.0	49.0	45.1	49.0
AFR	Guinea		47.0	0.0	47.0	43.2	47.0
AFR	Chad		37.0	0.0	37.0	34.0	37.0
AMR	Cuba	99.0	99.0	98.0	2.0	94.9	100.0
AMR	Nicaragua	99.0	99.0	98.0	2.0	94.9	100.0
AMR	Saint Vincent and the Grenadines	99.0	99.0	98.0	2.0	94.9	100.0
AMR	Uruguay	99.0	96.0	95.0	4.9	94.8	100.0
AMR	Panama	97.0	98.0	95.1	4.9	94.8	99.9
AMR	Antigua and Barbuda	95.0	99.0	94.1	5.9	94.8	100.0
AMR	Guyana	92.0	99.0	91.1	8.8	94.7	99.9
AMR	Saint Kitts and Nevis	98.0	93.0	91.1	8.7	94.6	99.9
AMR	Costa Rica	93.0	96.0	89.3	10.4	94.4	99.7
AMR	Jamaica	92.0	95.0	87.4	12.2	94.3	99.6
AMR	United States of America	95.0	92.0	87.4	12.2	94.3	99.6
AMR	Belize	95.0	90.0	85.5	14.0	94.1	99.5
AMR	Trinidad and Tobago	92.0	93.0	85.6	13.9	94.1	99.4
AMR	Chile	91.0	93.0	84.6	14.7	94.0	99.4
AMR	Honduras	85.0	96.0	81.6	17.8	93.9	99.4
AMR	Colombia	88.0	93.0	81.8	17.3	93.7	99.2
AMR	Argentina	89.0	89.0	79.2	19.6	93.3	98.8
AMR	Canada	87.0	90.0	78.3	20.4	93.2	98.7
AMR	Paraguay	83.0	92.0	76.4	22.3	93.0	98.6
AMR	Bahamas	82.0	90.0	73.8	24.4	92.6	98.2
AMR	Barbados	77.0	92.0	70.8	27.3	92.4	98.2
AMR	Dominica	92.0	77.0	70.8	27.3	92.4	98.2
AMR	El Salvador	87.0	85.0	74.0	24.1	92.4	98.1
AMR	Grenada	82.0	85.0	69.7	27.6	91.6	97.3
AMR	Guatemala	78.0	86.0	67.1	29.8	91.2	96.9
AMR	Saint Lucia	75.0	87.0	65.3	31.5	91.0	96.8

AMR	Brazil	54.0	91.0	49.1	46.7	89.7	95.9
AMR	Ecuador	76.0	81.0	61.6	33.9	89.7	95.4
AMR	Venezuela	13.0	96.0	12.5	84.0	89.2	96.5
AMR	Dominican Republic	60.0	86.0	51.6	42.8	88.4	94.4
AMR	Peru	66.0	83.0	54.8	39.4	88.3	94.2
AMR	Mexico	73.0	76.0	55.5	38.0	87.7	93.5
AMR	Bolivia	44.0	83.0	36.5	54.0	84.3	90.5
AMR	Suriname	58.0	76.0	44.1	45.8	84.0	89.9
AMR	Haiti	41.0	69.0	28.3	53.4	76.0	81.7
EMR	Bahrain	99.0	99.0	98.0	2.0	94.9	100.0
EMR	Morocco	99.0	99.0	98.0	2.0	94.9	100.0
EMR	Oman	99.0	99.0	98.0	2.0	94.9	100.0
EMR	United Arab Emirates	99.0	99.0	98.0	2.0	94.9	100.0
EMR	Iran	98.0	99.0	97.0	3.0	94.9	100.0
EMR	Qatar	95.0	99.0	94.1	5.9	94.8	100.0
EMR	Kuwait	94.0	99.0	93.1	6.9	94.7	99.9
EMR	Saudi Arabia	96.0	96.0	92.2	7.7	94.6	99.8
EMR	Jordan	96.0	93.0	89.3	10.4	94.4	99.7
EMR	Egypt	94.0	94.0	88.4	11.3	94.3	99.6
EMR	Tunisia	93.0	91.0	84.6	14.7	94.0	99.4
EMR	Libya	72.0	94.0	67.7	30.6	92.5	98.3
EMR	Iraq	86.0	85.0	73.1	24.8	92.3	97.9
EMR	Sudan	74.0	90.0	66.6	30.8	91.6	97.4
EMR	Djibouti	81.0	81.0	65.6	30.8	90.6	96.4
EMR	Lebanon	63.0	82.0	51.7	41.7	87.4	93.3
EMR	Pakistan	71.0	76.0	54.0	39.1	87.2	93.0
EMR	Syrian Arab Republic	54.0	67.0	36.2	48.6	79.1	84.8
EMR	Yemen	46.0	65.0	29.9	51.2	75.5	81.1
EMR	Afghanistan	39.0	64.0	25.0	53.1	72.5	78.0
EMR	Somalia		46.0	0.0	46.0	42.3	46.0
EUR	Hungary	99.0	99.0	98.0	2.0	94.9	100.0
EUR	Turkmenistan	99.0	99.0	98.0	2.0	94.9	100.0
EUR	Uzbekistan	99.0	99.0	98.0	2.0	94.9	100.0
EUR	Kazakhstan	98.0	99.0	97.0	3.0	94.9	100.0
EUR	Azerbaijan	97.0	98.0	95.1	4.9	94.8	99.9
EUR	Belarus	98.0	97.0	95.1	4.9	94.8	99.9
EUR	Russian Federation	97.0	98.0	95.1	4.9	94.8	99.9
EUR	Tajikistan	97.0	98.0	95.1	4.9	94.8	99.9
EUR	Andorra	95.0	99.0	94.1	5.9	94.8	100.0
EUR	Israel	96.0	98.0	94.1	5.8	94.7	99.9
EUR	Portugal	96.0	98.0	94.1	5.8	94.7	99.9
EUR	Slovakia	98.0	96.0	94.1	5.8	94.7	99.9
EUR	Kyrgyzstan	98.0	95.0	93.1	6.8	94.7	99.9
EUR	Spain	94.0	98.0	92.1	7.8	94.7	99.9
EUR	Georgia	97.0	95.0	92.2	7.7	94.6	99.9

EUR	Sweden	95.0	97.0	92.2	7.7	94.6	99.9
EUR	Albania	96.0	96.0	92.2	7.7	94.6	99.8
EUR	Armenia	96.0	96.0	92.2	7.7	94.6	99.8
EUR	Latvia	96.0	96.0	92.2	7.7	94.6	99.8
EUR	Luxembourg	90.0	99.0	89.1	10.8	94.6	99.9
EUR	Norway	95.0	96.0	91.2	8.6	94.6	99.8
EUR	Germany	93.0	97.0	90.2	9.6	94.5	99.8
EUR	Denmark	90.0	97.0	87.3	12.4	94.3	99.7
EUR	Republic of Moldova	95.0	93.0	88.4	11.3	94.3	99.7
EUR	Iceland	95.0	92.0	87.4	12.2	94.3	99.6
EUR	Finland	93.0	94.0	87.4	12.2	94.2	99.6
EUR	Lithuania	93.0	94.0	87.4	12.2	94.2	99.6
EUR	Slovenia	94.0	93.0	87.4	12.2	94.2	99.6
EUR	Malta	95.0	91.0	86.5	13.1	94.2	99.6
EUR	Poland	92.0	94.0	86.5	13.0	94.2	99.5
EUR	Switzerland	90.0	95.0	85.5	14.0	94.1	99.5
EUR	Turkey	88.0	96.0	84.5	15.0	94.1	99.5
EUR	Croatia	95.0	89.0	84.6	14.9	94.0	99.5
EUR	Czechia	84.0	97.0	81.5	18.0	94.0	99.5
EUR	Greece	83.0	97.0	80.5	19.0	93.9	99.5
EUR	Belgium	85.0	96.0	81.6	17.8	93.9	99.4
EUR	Estonia	90.0	93.0	83.7	15.6	93.9	99.3
EUR	Netherlands	90.0	93.0	83.7	15.6	93.9	99.3
EUR	Austria	84.0	96.0	80.6	18.7	93.8	99.4
EUR	Bulgaria	87.0	94.0	81.8	17.4	93.7	99.2
EUR	Italy	88.0	92.0	81.0	18.1	93.5	99.0
EUR	United Kingdom of Great Britain and Northern Ireland	87.0	92.0	80.0	18.9	93.4	99.0
EUR	North Macedonia	94.0	83.0	78.0	21.0	93.4	99.0
EUR	Ukraine	92.0	86.0	79.1	19.8	93.3	98.9
EUR	Cyprus	88.0	90.0	79.2	19.6	93.3	98.8
EUR	Serbia	91.0	86.0	78.3	20.5	93.2	98.7
EUR	France	83.0	90.0	74.7	23.6	92.7	98.3
EUR	Monaco	79.0	88.0	69.5	28.0	91.8	97.5
EUR	San Marino	79.0	86.0	67.9	29.1	91.3	97.1
EUR	Romania	76.0	86.0	65.4	31.3	90.9	96.6
EUR	Montenegro	86.0	58.0	49.9	44.2	88.1	94.1
EUR	Bosnia and Herzegovina	76.0	69.0	52.4	40.1	86.7	92.6
EUR	Ireland		92.0	0.0	92.0	84.6	92.0
SEAR	Maldives	99.0	99.0	98.0	2.0	94.9	100.0
SEAR	Sri Lanka	99.0	99.0	98.0	2.0	94.9	100.0
SEAR	Democratic People's Republic of Korea	98.0	99.0	97.0	3.0	94.9	100.0

SEAR	Bangladesh	95.0	97.0	92.2	7.7	94.6	99.9
SEAR	Thailand	87.0	99.0	86.1	13.7	94.5	99.9
SEAR	Bhutan	92.0	97.0	89.2	10.5	94.5	99.8
SEAR	India	84.0	90.0	75.6	22.8	92.8	98.4
SEAR	Nepal	76.0	90.0	68.4	29.2	91.8	97.6
SEAR	Indonesia	71.0	90.0	63.9	33.2	91.2	97.1
SEAR	Myanmar	80.0	83.0	66.4	30.2	90.9	96.6
SEAR	Timor-Leste	80.0	77.0	61.6	33.8	89.6	95.4
WPR	Niue	99.0	99.0	98.0	2.0	94.9	100.0
WPR	Tonga	99.0	99.0	98.0	2.0	94.9	100.0
WPR	China	98.0	99.0	97.0	3.0	94.9	100.0
WPR	Mongolia	98.0	99.0	97.0	3.0	94.9	100.0
WPR	Cook Islands	98.0	98.0	96.0	3.9	94.8	100.0
WPR	Brunei Darussalam	98.0	97.0	95.1	4.9	94.8	99.9
WPR	Republic of Korea	96.0	97.0	93.1	6.8	94.7	99.9
WPR	Japan	93.0	97.0	90.2	9.6	94.5	99.8
WPR	Fiji	94.0	96.0	90.2	9.5	94.5	99.8
WPR	Nauru	95.0	95.0	90.3	9.5	94.5	99.8
WPR	Viet Nam	92.0	97.0	89.2	10.5	94.5	99.8
WPR	Australia	94.0	95.0	89.3	10.4	94.4	99.7
WPR	Tuvalu	92.0	95.0	87.4	12.2	94.3	99.6
WPR	New Zealand	90.0	93.0	83.7	15.6	93.9	99.3
WPR	Palau	88.0	93.0	81.8	17.3	93.7	99.2
WPR	Singapore	84.0	95.0	79.8	19.4	93.7	99.2
WPR	Malaysia	87.0	93.0	80.9	18.2	93.6	99.1
WPR	Kiribati	91.0	81.0	73.7	24.6	92.6	98.3
WPR	Cambodia	82.0	84.0	68.9	28.2	91.4	97.1
WPR	Marshall Islands	64.0	83.0	53.1	40.8	88.0	93.9
WPR	Solomon Islands	54.0	84.0	45.4	47.3	86.6	92.6
WPR	Micronesia	52.0	76.0	39.5	49.0	82.6	88.5
WPR	Lao People's Democratic Republic	57.0	72.0	41.0	46.9	82.2	88.0
WPR	Philippines	40.0	73.0	29.2	54.6	78.0	83.8
WPR	Vanuatu		80.0	0.0	80.0	73.6	80.0
WPR	Samoa	44.0	58.0	25.5	51.0	71.1	76.5
WPR	Papua New Guinea	20.0	38.0	7.6	42.8	46.6	50.4

AFR: African region; AMR: American region; EMR: Eastern Mediterranean region; EUR: European region; SEAR: South-East Asia region; WPR: Western pacific region

Table S2: Pertussis vaccination coverage and pertussis protection in countries of the world in 2019: 1) vaccination coverage with the first (DTP1), second (DTP2) and third (DTP3) doses of pertussis vaccine ; 2) vaccination coverage with one, two and three doses of DTP vaccine in the target vaccination population; 3) pertussis protection in terms of prevalence of individuals with vaccine-induced pertussis protection in the target vaccination population; and 4) vaccination coverage with one, two or three doses of DTP vaccine

		Vaccination corage (%)			Vaccination coverage (%)				Vaccination coverage (%) with one, two or three doses
Region	Country name	DTP1 vaccine	DTP2* vaccine	DTP3 vaccine	3 doses	2 doses	1 dose	Pertussis protection (%)	
AFR	Algeria	96.0	93.5	91.0	81.7	17.2	1.1	82.5	100.0
AFR	Angola	67.0	62.0	57.0	23.7	44.0	26.9	69.7	94.6
AFR	Benin	84.0	80.0	76.0	51.1	38.6	9.5	78.3	99.2
AFR	Botswana	98.0	96.5	95.0	89.8	9.8	0.3	83.2	100.0
AFR	Burkina Faso	95.0	93.0	91.0	80.4	18.2	1.3	82.4	100.0
AFR	Burundi	97.0	95.0	93.0	85.7	13.6	0.7	82.9	100.0
AFR	Cabo Verde	96.0	96.0	96.0	88.5	11.1	0.5	83.1	100.0
AFR	Cameroon	75.0	71.0	67.0	35.7	44.0	17.9	74.4	97.6
AFR	Central African Republic	69.0	58.0	47.0	18.8	43.3	31.0	67.4	93.1
AFR	Chad	65.0	57.5	50.0	18.7	42.6	31.3	66.9	92.6
AFR	Comoros	96.0	93.5	91.0	81.7	17.2	1.1	82.5	100.0
AFR	Congo	82.0	80.5	79.0	52.1	37.9	9.2	78.4	99.3
AFR	Côte d'Ivoire	98.0	91.0	84.0	74.9	23.2	1.9	81.9	100.0
AFR	Democratic Republic of the Congo	66.0	61.5	57.0	23.1	43.9	27.4	69.4	94.4
AFR	Equatorial Guinea	77.0	65.0	53.0	26.5	45.7	24.0	71.6	96.2
AFR	Eritrea	97.0	96.0	95.0	88.5	11.1	0.5	83.1	100.0
AFR	Eswatini	96.0	93.0	90.0	80.4	18.3	1.3	82.4	100.0
AFR	Ethiopia	80.0	74.5	69.0	41.1	42.8	14.5	76.1	98.4
AFR	Gabon	77.0	73.5	70.0	39.6	43.1	15.5	75.6	98.2
AFR	Gambia	93.0	90.5	88.0	74.1	23.4	2.4	81.7	99.9
AFR	Ghana	97.0	97.0	97.0	91.3	8.5	0.3	83.3	100.0
AFR	Guinea	62.0	54.5	47.0	15.9	40.9	34.1	64.9	90.8
AFR	Guinea-Bissau	85.0	84.5	84.0	60.3	33.2	6.1	79.8	99.6
AFR	Kenya	97.0	94.5	92.0	84.3	14.8	0.8	82.7	100.0
AFR	Lesotho	92.0	89.5	87.0	71.6	25.3	2.9	81.4	99.9
AFR	Liberia	94.0	84.0	74.0	58.4	35.4	5.9	79.8	99.8
AFR	Madagascar	85.0	82.0	79.0	55.1	36.4	7.9	79.0	99.4
AFR	Malawi	97.0	96.0	95.0	88.5	11.1	0.5	83.1	100.0
AFR	Mali	82.0	79.5	77.0	50.2	39.0	10.0	78.1	99.2
AFR	Mauritania	89.0	85.0	81.0	61.3	32.8	5.6	80.0	99.7

AFR	Mauritius	97.0	96.5	96.0	89.9	9.8	0.4	83.2	100.0
AFR	Mozambique	93.0	90.5	88.0	74.1	23.4	2.4	81.7	99.9
AFR	Namibia	92.0	89.5	87.0	71.6	25.3	2.9	81.4	99.9
AFR	Niger	92.0	86.5	81.0	64.5	30.8	4.5	80.5	99.8
AFR	Nigeria	65.0	61.0	57.0	22.6	43.7	27.9	69.0	94.1
AFR	Rwanda	99.0	98.5	98.0	95.6	4.4	0.1	83.7	100.0
AFR	Sao Tome and Principe	97.0	96.0	95.0	88.5	11.1	0.5	83.1	100.0
AFR	Senegal	97.0	95.0	93.0	85.7	13.6	0.7	82.9	100.0
AFR	Seychelles	99.0	99.0	99.0	97.0	2.9	0.0	83.8	100.0
AFR	Sierra Leone	95.0	95.0	95.0	85.7	13.5	0.7	82.9	100.0
AFR	South Africa	84.0	80.5	77.0	52.1	38.1	9.1	78.4	99.3
AFR	South Sudan	51.0	50.0	49.0	12.5	37.5	37.5	61.5	87.5
AFR	Togo	90.0	87.0	84.0	65.8	29.7	4.4	80.7	99.8
AFR	Uganda	99.0	96.0	93.0	88.4	11.2	0.4	83.1	100.0
AFR	United Republic of Tanzania	91.0	90.0	89.0	72.9	24.3	2.7	81.5	99.9
AFR	Zambia	94.0	91.0	88.0	75.3	22.5	2.1	81.8	99.9
AFR	Zimbabwe	94.0	92.0	90.0	77.8	20.4	1.7	82.1	100.0
AMR	Antigua and Barbuda	99.0	97.0	95.0	91.2	8.5	0.2	83.3	100.0
AMR	Argentina	91.0	88.5	86.0	69.3	27.1	3.5	81.1	99.9
AMR	Bahamas	91.0	88.5	86.0	69.3	27.1	3.5	81.1	99.9
AMR	Barbados	92.0	91.0	90.0	75.3	22.4	2.2	81.8	99.9
AMR	Belize	99.0	98.5	98.0	95.6	4.4	0.1	83.7	100.0
AMR	Bolivia	81.0	78.0	75.0	47.4	40.3	11.3	77.5	99.0
AMR	Brazil	81.0	77.0	73.0	45.5	41.1	12.2	77.1	98.8
AMR	Canada	94.0	92.5	91.0	79.1	19.3	1.5	82.2	100.0
AMR	Chile	99.0	97.5	96.0	92.7	7.2	0.2	83.5	100.0
AMR	Colombia	92.0	92.0	92.0	77.9	20.3	1.8	82.1	99.9
AMR	Costa Rica	96.0	95.5	95.0	87.1	12.3	0.6	83.0	100.0
AMR	Cuba	99.0	99.0	99.0	97.0	2.9	0.0	83.8	100.0
AMR	Dominica	99.0	99.0	99.0	97.0	2.9	0.0	83.8	100.0
AMR	Dominican Republic	99.0	94.0	89.0	82.8	16.4	0.8	82.6	100.0
AMR	Ecuador	85.0	85.0	85.0	61.4	32.5	5.7	80.0	99.7
AMR	El Salvador	82.0	81.5	81.0	54.1	36.9	8.4	78.8	99.4
AMR	Grenada	96.0	94.0	92.0	83.0	16.0	1.0	82.6	100.0
AMR	Guatemala	92.0	88.5	85.0	69.2	27.2	3.4	81.1	99.9
AMR	Guyana	99.0	99.0	99.0	97.0	2.9	0.0	83.8	100.0
AMR	Haiti	75.0	63.0	51.0	24.1	45.3	26.0	70.5	95.5
AMR	Honduras	90.0	88.5	87.0	69.3	27.1	3.5	81.1	99.9
AMR	Jamaica	97.0	96.5	96.0	89.9	9.8	0.4	83.2	100.0
AMR	Mexico	84.0	83.0	82.0	57.2	35.1	7.2	79.3	99.5
AMR	Nicaragua	99.0	98.5	98.0	95.6	4.4	0.1	83.7	100.0
AMR	Panama	96.0	92.0	88.0	77.7	20.6	1.6	82.1	100.0

AMR	Paraguay	88.0	87.0	86.0	65.8	29.5	4.4	80.6	99.8
AMR	Peru	97.0	92.5	88.0	79.0	19.6	1.4	82.3	100.0
AMR	Saint Kitts and Nevis	99.0	97.5	96.0	92.7	7.2	0.2	83.5	100.0
AMR	Saint Lucia	99.0	95.5	92.0	87.0	12.5	0.5	83.0	100.0
AMR	Saint Vincent and the Grenadines	99.0	98.0	97.0	94.1	5.8	0.1	83.6	100.0
AMR	Suriname	81.0	79.0	77.0	49.3	39.4	10.4	77.9	99.1
AMR	Trinidad and Tobago	97.0	95.0	93.0	85.7	13.6	0.7	82.9	100.0
AMR	United States of America	97.0	95.5	94.0	87.1	12.4	0.6	83.0	100.0
AMR	Uruguay	99.0	96.5	94.0	89.8	9.9	0.3	83.2	100.0
AMR	Venezuela	85.0	74.5	64.0	40.5	43.8	14.3	76.2	98.6
EMR	Afghanistan	73.0	69.5	66.0	33.5	44.3	19.4	73.7	97.2
EMR	Bahrain	99.0	99.0	99.0	97.0	2.9	0.0	83.8	100.0
EMR	Djibouti	90.0	87.5	85.0	66.9	28.8	4.1	80.8	99.8
EMR	Egypt	96.0	95.5	95.0	87.1	12.3	0.6	83.0	100.0
EMR	Iran	99.0	99.0	99.0	97.0	2.9	0.0	83.8	100.0
EMR	Iraq	93.0	88.5	84.0	69.1	27.4	3.4	81.1	99.9
EMR	Jordan	90.0	89.5	89.0	71.7	25.2	3.0	81.4	99.9
EMR	Kuwait	99.0	95.0	91.0	85.6	13.8	0.6	82.9	100.0
EMR	Lebanon	96.0	89.5	83.0	71.3	25.9	2.7	81.5	99.9
EMR	Libya	74.0	73.5	73.0	39.7	43.0	15.5	75.6	98.1
EMR	Morocco	99.0	99.0	99.0	97.0	2.9	0.0	83.8	100.0
EMR	Oman	99.0	99.0	99.0	97.0	2.9	0.0	83.8	100.0
EMR	Pakistan	86.0	80.5	75.0	51.9	38.3	9.1	78.5	99.3
EMR	Qatar	99.0	98.5	98.0	95.6	4.4	0.1	83.7	100.0
EMR	Saudi Arabia	96.0	96.0	96.0	88.5	11.1	0.5	83.1	100.0
EMR	Somalia	52.0	47.0	42.0	10.3	35.2	39.8	59.2	85.2
EMR	Sudan	97.0	95.0	93.0	85.7	13.6	0.7	82.9	100.0
EMR	Syrian Arab Republic	72.0	63.0	54.0	24.5	44.8	26.0	70.4	95.2
EMR	Tunisia	97.0	94.5	92.0	84.3	14.8	0.8	82.7	100.0
EMR	United Arab Emirates	99.0	99.0	99.0	97.0	2.9	0.0	83.8	100.0
EMR	Yemen	81.0	77.0	73.0	45.5	41.1	12.2	77.1	98.8
EUR	Albania	99.0	99.0	99.0	97.0	2.9	0.0	83.8	100.0
EUR	Andorra	99.0	99.0	99.0	97.0	2.9	0.0	83.8	100.0
EUR	Armenia	96.0	94.0	92.0	83.0	16.0	1.0	82.6	100.0
EUR	Austria	90.0	87.5	85.0	66.9	28.8	4.1	80.8	99.8
EUR	Azerbaijan	96.0	95.0	94.0	85.7	13.6	0.7	82.9	100.0
EUR	Belarus	98.0	98.0	98.0	94.1	5.8	0.1	83.6	100.0
EUR	Belgium	99.0	98.5	98.0	95.6	4.4	0.1	83.7	100.0
EUR	Bosnia and Herzegovina	89.0	81.0	73.0	52.6	38.3	8.5	78.7	99.4

EUR	Bulgaria	94.0	93.0	92.0	80.4	18.2	1.4	82.4	100.0
EUR	Croatia	98.0	96.0	94.0	88.4	11.1	0.4	83.1	100.0
EUR	Cyprus	98.0	97.0	96.0	91.3	8.5	0.3	83.3	100.0
EUR	Czechia	99.0	98.0	97.0	94.1	5.8	0.1	83.6	100.0
EUR	Denmark	97.0	97.0	97.0	91.3	8.5	0.3	83.3	100.0
EUR	Estonia	92.0	91.5	91.0	76.6	21.4	2.0	82.0	99.9
EUR	Finland	98.0	94.5	91.0	84.3	15.0	0.8	82.8	100.0
EUR	France	99.0	97.5	96.0	92.7	7.2	0.2	83.5	100.0
EUR	Georgia	99.0	96.5	94.0	89.8	9.9	0.3	83.2	100.0
EUR	Germany	98.0	95.5	93.0	87.0	12.4	0.5	83.0	100.0
EUR	Greece	99.0	99.0	99.0	97.0	2.9	0.0	83.8	100.0
EUR	Hungary	99.0	99.0	99.0	97.0	2.9	0.0	83.8	100.0
EUR	Iceland	97.0	94.0	91.0	83.0	16.1	0.9	82.6	100.0
EUR	Ireland	98.0	96.0	94.0	88.4	11.1	0.4	83.1	100.0
EUR	Israel	99.0	98.5	98.0	95.6	4.4	0.1	83.7	100.0
EUR	Italy	95.0	95.0	95.0	85.7	13.5	0.7	82.9	100.0
EUR	Kazakhstan	99.0	98.0	97.0	94.1	5.8	0.1	83.6	100.0
EUR	Kyrgyzstan	99.0	97.0	95.0	91.2	8.5	0.2	83.3	100.0
EUR	Latvia	99.0	99.0	99.0	97.0	2.9	0.0	83.8	100.0
EUR	Lithuania	96.0	94.0	92.0	83.0	16.0	1.0	82.6	100.0
EUR	Luxembourg	99.0	99.0	99.0	97.0	2.9	0.0	83.8	100.0
EUR	Malta	98.0	98.0	98.0	94.1	5.8	0.1	83.6	100.0
EUR	Monaco	99.0	99.0	99.0	97.0	2.9	0.0	83.8	100.0
EUR	Montenegro	94.0	90.0	86.0	72.8	24.6	2.6	81.6	99.9
EUR	Netherlands	98.0	96.0	94.0	88.4	11.1	0.4	83.1	100.0
EUR	North Macedonia	98.0	95.0	92.0	85.7	13.7	0.6	82.9	100.0
EUR	Norway	99.0	98.0	97.0	94.1	5.8	0.1	83.6	100.0
EUR	Poland	98.0	96.5	95.0	89.8	9.8	0.3	83.2	100.0
EUR	Portugal	99.0	99.0	99.0	97.0	2.9	0.0	83.8	100.0
EUR	Republic of Moldova	91.0	91.0	91.0	75.4	22.4	2.2	81.8	99.9
EUR	Romania	94.0	91.0	88.0	75.3	22.5	2.1	81.8	99.9
EUR	Russian Federation	97.0	97.0	97.0	91.3	8.5	0.3	83.3	100.0
EUR	San Marino	90.0	89.0	88.0	70.5	26.2	3.2	81.3	99.9
EUR	Serbia	99.0	98.0	97.0	94.1	5.8	0.1	83.6	100.0
EUR	Slovakia	99.0	98.0	97.0	94.1	5.8	0.1	83.6	100.0
EUR	Slovenia	98.0	96.5	95.0	89.8	9.8	0.3	83.2	100.0
EUR	Spain	98.0	97.0	96.0	91.3	8.5	0.3	83.3	100.0
EUR	Sweden	98.0	98.0	98.0	94.1	5.8	0.1	83.6	100.0
EUR	Switzerland	98.0	97.0	96.0	91.3	8.5	0.3	83.3	100.0
EUR	Tajikistan	98.0	97.5	97.0	92.7	7.1	0.2	83.5	100.0
EUR	Turkey	99.0	99.0	99.0	97.0	2.9	0.0	83.8	100.0
EUR	Turkmenistan	99.0	99.0	99.0	97.0	2.9	0.0	83.8	100.0
EUR	Ukraine	92.0	86.0	80.0	63.3	31.6	4.8	80.4	99.8

EUR	United Kingdom of Great Britain and Northern Ireland	97.0	95.0	93.0	85.7	13.6	0.7	82.9	100.0
EUR	Uzbekistan	96.0	96.0	96.0	88.5	11.1	0.5	83.1	100.0
SEAR	Bangladesh	99.0	98.5	98.0	95.6	4.4	0.1	83.7	100.0
SEAR	Bhutan	99.0	98.0	97.0	94.1	5.8	0.1	83.6	100.0
SEAR	Democratic People's Republic of Korea	98.0	97.5	97.0	92.7	7.1	0.2	83.5	100.0
SEAR	India	94.0	92.5	91.0	79.1	19.3	1.5	82.2	100.0
SEAR	Indonesia	90.0	87.5	85.0	66.9	28.8	4.1	80.8	99.8
SEAR	Maldives	99.0	99.0	99.0	97.0	2.9	0.0	83.8	100.0
SEAR	Myanmar	93.0	91.5	90.0	76.6	21.4	2.0	82.0	99.9
SEAR	Nepal	96.0	94.5	93.0	84.4	14.8	0.8	82.7	100.0
SEAR	Sri Lanka	99.0	99.0	99.0	97.0	2.9	0.0	83.8	100.0
SEAR	Thailand	99.0	98.0	97.0	94.1	5.8	0.1	83.6	100.0
SEAR	Timor-Leste	92.0	87.5	83.0	66.8	29.0	4.0	80.8	99.8
WPR	Australia	98.0	96.5	95.0	89.8	9.8	0.3	83.2	100.0
WPR	Brunei Darussalam	99.0	99.0	99.0	97.0	2.9	0.0	83.8	100.0
WPR	Cambodia	94.0	93.0	92.0	80.4	18.2	1.4	82.4	100.0
WPR	China	99.0	99.0	99.0	97.0	2.9	0.0	83.8	100.0
WPR	Cook Islands	99.0	98.5	98.0	95.6	4.4	0.1	83.7	100.0
WPR	Fiji	99.0	99.0	99.0	97.0	2.9	0.0	83.8	100.0
WPR	Japan	99.0	98.5	98.0	95.6	4.4	0.1	83.7	100.0
WPR	Kiribati	99.0	98.0	97.0	94.1	5.8	0.1	83.6	100.0
WPR	Lao People's Democratic Republic	73.0	70.5	68.0	35.0	44.1	18.4	74.2	97.5
WPR	Malaysia	99.0	98.5	98.0	95.6	4.4	0.1	83.7	100.0
WPR	Marshall Islands	96.0	87.5	79.0	66.4	29.9	3.6	80.9	99.9
WPR	Micronesia	97.0	87.5	78.0	66.2	30.2	3.5	80.9	99.9
WPR	Mongolia	99.0	98.5	98.0	95.6	4.4	0.1	83.7	100.0
WPR	Nauru	99.0	97.5	96.0	92.7	7.2	0.2	83.5	100.0
WPR	New Zealand	94.0	93.0	92.0	80.4	18.2	1.4	82.4	100.0
WPR	Niue	99.0	99.0	99.0	97.0	2.9	0.0	83.8	100.0
WPR	Palau	99.0	98.0	97.0	94.1	5.8	0.1	83.6	100.0
WPR	Papua New Guinea	44.0	39.5	35.0	6.1	28.4	43.5	52.6	78.0
WPR	Philippines	66.0	65.5	65.0	28.1	44.4	23.4	71.6	95.9
WPR	Republic of Korea	98.0	98.0	98.0	94.1	5.8	0.1	83.6	100.0
WPR	Samoa	86.0	72.0	58.0	35.9	45.8	16.6	75.3	98.4
WPR	Singapore	98.0	97.0	96.0	91.3	8.5	0.3	83.3	100.0
WPR	Solomon Islands	98.0	96.0	94.0	88.4	11.1	0.4	83.1	100.0
WPR	Tonga	99.0	99.0	99.0	97.0	2.9	0.0	83.8	100.0
WPR	Tuvalu	99.0	95.5	92.0	87.0	12.5	0.5	83.0	100.0
WPR	Vanuatu	95.0	92.5	90.0	79.1	19.4	1.5	82.2	100.0
WPR	Viet Nam	96.0	92.5	89.0	79.0	19.5	1.5	82.2	100.0

* Vaccination coverage with DTP2 vaccine estimated from: (DTP1 + DTP3)/2

AFR: African region; AMR: American region; EMR: Eastern Mediterranean region; EUR: European region; SEAR: South-East Asia region; WPR: Western pacific region
